# SpaceXray: Feasibility and Diagnostic Capabilities of On-Orbit Medical Radiography

**DOI:** 10.1148/radiol.260258

**Published:** 2026-07-14

**Authors:** Sheyna E. Gifford, Michael Pohlen, Adam S. Wang, David J. Lerner, Anna Wadhwa, Michael Cairnie, Jeanne Walter, Karim S. Karim, Steven Tilley, Amol Karnick, Marissa A. Rosenberg, Lonnie G. Petersen

**Affiliations:** ^1^Department of Aerospace Medicine, Division of Public Health, Infectious Diseases, and Occupational Medicine, Mayo Clinic Rochester, 200 1st St SW, Rochester, MN 55905; ^2^Department of Radiology, University of California, San Diego, La Jolla, Calif; ^3^Departments of Radiology and Electrical Engineering, Stanford University, Stanford, Calif; ^4^Radiology Logistics Consultants, Seattle, Wash; ^5^Department of Aeronautics and Astronautics, Massachusetts Institute of Technology, Cambridge, Mass; ^6^Health Sciences and Technology, Harvard Medical School/Institute for Medical Engineering and Science at Massachusetts Institute of Technology, Boston, Mass; ^7^MinXray, Northbrook, Ill; ^8^KA Imaging, Waterloo, Ontario, Canada; ^9^University of Waterloo, Waterloo, Ontario, Canada; ^10^Space Exploration Technologies, Hawthorne, Calif

## Abstract

**Background:**

To support crew health during spaceflight, in-flight imaging must extend beyond the capabilities of portable ultrasound (US). Digital radiography may provide improved diagnostic quality and enable equipment monitoring through nondestructive testing.

**Purpose:**

To demonstrate the feasibility of a commercial-off-the-shelf (COTS) radiography system for on-orbit imaging applications.

**Materials and Methods:**

In a prospective study, a portable, digital, COTS radiography system was evaluated during the 3.5-day Fram2 polar orbital flight. Anatomic and equipment radiographs were obtained preflight and in-flight by crew participants with 4 hours of training. Radiographs were evaluated by independent radiologists on overall image quality, spatial resolution, contrast resolution, and positioning (Likert scores of 1–5). Preflight radiographs were compared with in-flight radiographs using the Wilcoxon rank sum test. Hardware testing and crew surveys were conducted postflight; survey responses were thematically analyzed due to small sample size.

**Results:**

Three crewmembers participated (mean age, 42.8 years ± 13.9 [SD]; two women). In-flight and preflight anatomic radiographs (seven each) demonstrated no evidence of differences in overall image quality (mean score, 4.86 ± 0.26 vs 5.0 ± 0, respectively; *P* > .99), spatial resolution (mean score, 4.86 ± 0.26 vs 5.00 ± 0; *P* = .46), or contrast resolution (mean score, 4.86 ± 0.26 vs 5.00 ± 0; *P* = .46). However, for central radiographs (chest, abdomen, pelvis; 12 of 14 images), image positioning was worse in-flight than preflight (mean score, 4.07 ± 0.72 vs 4.95 ± 0.13, respectively; *P* = .02). In-flight nondestructive testing enabled visualization of internal equipment components to the submillimeter scale. The radiography system functioned nominally postflight despite minor re-entry damage. Crew surveys rated equipment and protocols easy to use.

**Conclusion:**

By acquiring the first human radiographs in space, this study demonstrated the feasibility of on-orbit radiography, expanded diagnostic capabilities for crew health and equipment evaluation, and identified operational standardization gaps and image-aligned challenges in microgravity.

© The Author(s) 2026. Published by the Radiological Society of North America under a CC BY 4.0 license.

*Supplemental material is available for this article.*

See also the editorial by Abbara and McMillan in this issue.

## Graphical abstract

**Figure:**
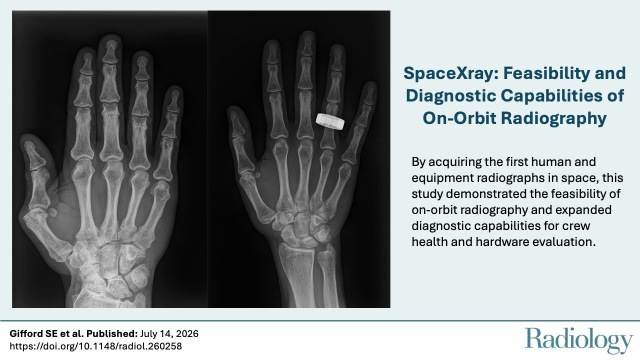

**Summary**

By acquiring the first human and equipment radiographs in space, this study demonstrated the feasibility of on-orbit radiography and expanded diagnostic capabilities for crew health and hardware evaluation.

**Key Results**

■ Minimally trained nonmedical crewmembers (*n* = 3) acquired diagnostically adequate anatomic and equipment-monitoring radiographs during orbital flight.
■ Preflight and in-flight phantom radiographs demonstrated no evidence of differences in overall image quality (*P* > .99), spatial resolution (*P* = .46), or contrast resolution (*P* = .46); however, image positioning for central body parts was worse in-flight than preflight (*P* = .02).
■ Crew surveys identified alignment and positioning, including of the subject, x-ray source, and detector, as the primary operational challenges, while equipment and protocols were rated easy to use.

## Introduction

For more than 4 decades, the only reliable medical imaging modality in orbit was ultrasound (US). As spaceflight missions increase in duration and distance, the risk of adverse medical events rises and the limitations of US as a lone imaging modality—operator dependence, reliance on acoustic windows and a soundwave-transmitting medium, and substantial training requirements—become less acceptable (1–4). A more ideal deep space imaging system would be usable by any crewmember with minimal training, require no consumables such as film screens or US gel, and support multiple mission-relevant medical and nonmedical operations.

Radiography offers superior diagnostic and management capabilities compared with US for up to 22% of National Aeronautics and Space Administration (NASA)–identified high-risk medical conditions for exploration-class missions (5,6). Because many urgent and emergent conditions are best or only diagnosed with radiography, ultraportable radiography systems equipped with remote interpretation capability, clinical decision support, and artificial intelligence are widely used in resource-limited communities on Earth (6–8). Digital radiography eliminates film use and reduces operator training requirements with automatic setting adjustments and laser positioning guides (9,10). Furthermore, high-spatial-resolution x‐ray imaging is routinely used to identify structural damage at microscales (11,12). Via in situ nondestructive testing (NDT), digital radiography could improve operational safety by characterizing structural anomalies or damage and assessing in-space manufacturing quality (11–15). A spaceflight-ready radiography system would have profound implications not only for crew health, but also for mission-critical nonmedical tasks; yet before this work, to our knowledge, digital radiography has never been demonstrated on-orbit for NDT or human physiology.

The purpose of this study was to demonstrate the feasibility of a portable, digital, commercial-off-the-shelf (COTS) radiography system for on-orbit anatomic and equipment imaging applications. The operational feasibility of ultraportable radiography in altered microgravity was first demonstrated by Lerner et al (16) in 2022. The Digital Ultraportable X-ray for Space, or DUXS, experiment used a COTS, battery-powered, digital x-ray device to take diagnostically adequate human anatomic and NDT images during lunar gravity (1/6*g*), Martian gravity (1/3*g*), and microgravity intervals of parabolic flight (16). We hypothesized that nonmedical crewmembers could acquire diagnostically adequate images using a similar portable digital radiography system during orbital flight.

## Materials and Methods

### Radiography System

This prospective study used a COTS, U.S. Food and Drug Administration (FDA)–cleared, ultraportable, wireless digital x-ray generator (Impact Wireless; MinXray). X-rays were detected with an FDA-cleared flat panel detector capable of single-exposure spectral dual-energy subtraction (Reveal 35C; KA Imaging) (17). This detector was selected for its potential to quantify areal bone mineral density (aBMD) in flight as well as for other diagnostic advantages (18–22). Full technical specifications of the x-ray generator and detector are provided in Appendix S1 and Tables S1 and S2.

All hardware underwent and passed system impact testing and compatibility testing for the Dragon spacecraft. SpaceX personnel undertook inspection; functional testing; vibration, temperature, and vacuum exposure for altitude simulation; electromagnetic interference testing; and external short-circuit and overcharge testing. The COTS x-ray generator and detector used for the SpaceXray study were loaned by the U.S. distributor and Canadian manufacturer, respectively, and returned at the conclusion of the Fram2 mission. Launch costs and hardware certification were provided by SpaceX at no cost to the study. Authors who were not employees of or consultants for these industries retained control over the inclusion of any data or information that might present a conflict of interest for authors who were employees of or consultants for these industries.

Research procedures were conducted in accordance with the guidelines of the Mayo Clinic institutional review board committee and were approved by the same committee (protocol: SpaceXray, version 25–000359). Written informed consent was obtained from the crew by SpaceX on behalf of all Fram2 investigators using their internal informed consent procedure. No direct or upfront industry funding was provided for the SpaceXray research study. The overall Fram2 mission was supported by the Translational Research Institute for Space Health, or TRISH, and SpaceX.

### Orbital Flight

The Fram2 mission launched on March 31, 2025, on a SpaceX Falcon 9 rocket. Four crewmembers aboard the Dragon spacecraft entered into a 90° polar orbit at 425–450 km above sea level. Splashdown took place in the Pacific Ocean on April 4, 2025 for a total mission duration of approximately 3 days 14 hours.

### Participants

Demographics of all four Fram2 crewmembers are shown in Table 1. Three of the four Fram2 astronauts opted to participate in the study. Inclusion criteria consisted of being a Fram2 flight crewmember, completion of equipment training, and age of 18–85 years. All three participants were confirmed eligible and included. All underwent imaging preflight. Only two crewmembers underwent imaging in flight due to operational time constraints. All three underwent imaging with dual-energy x-ray absorptiometry pre- and postflight.

**Table 1:** Demographic Characteristics of Fram2 Crewmembers

Characteristic	Metric
No. of study participants	3
No. of women	2
Age at time of launch (y)*	42.8 ± 13.9
Race/ethnicity	
Asian	1
White	3

Note.—Of the four Fram2 crewmembers, three participated and 50% were women. Further demographics of the three participating members are not provided separately because the small sample size could be potentially identifying.

* Data are mean age ± SD.

### Preflight Protocol

A train-the-trainer model was used in which MinXray and KA Imaging personnel provided approximately 2 hours of in-person training to SpaceX personnel on system operation and subject positioning. SpaceX personnel subsequently trained the Fram2 crew over two sessions totaling 4 hours, including image acquisition. Control phantom and human radiographs were acquired with dosimetry during preflight training. A spatial and contrast resolution phantom and Fenix 7 smartwatch (Garmin) used for NDT were secured to the detector using hook and loop fasteners with adhesive backing. The radiography quality control phantom (R/F QC Phantom 07–647; Supertech) was selected for its low mass, ease of use, and features including high-contrast meshes (20–100 lines per inch), low-contrast targets (2–8 mm diameter), and a 2-mm copper attenuator to simulate attenuation of an average adult.

Preflight, the crew acquired a full set of images identical to the planned in-flight protocol: hand, forearm, abdomen, and pelvis radiographs (one each) in one crewmember, followed by three chest and chest aBMD radiographs (*n* = 1–3). These were obtained at SpaceX. Dual-energy x-ray absorptiometry scans (Horizon W S/N300912M, Hologic; *n* = 3) were obtained at Houston Methodist Hospital. As the standard 72-inch distance for chest imaging was not achievable in-flight, all images were taken with a source-image distance of 130 cm between the generator and detector. Imaging protocols, including source-image difference, were identical across all examinations to ensure consistency.

### In-Flight Protocol

The day after launch (hereafter, L+1), in-flight radiographs of the phantom, a smartwatch, a hand, and a forearm (one exposure each) were obtained by the crew over 60 minutes of flight time. The initial protocol for day 3 after launch (hereafter, L+3) was for the crew to take five radiographs per participant: chest, abdomen, and pelvis; chest aBMD; and an air aBMD exposure with no physiology required for postprocessing. Due to operational time constraints and on-orbit alignment challenges, a total of five radiographs were obtained and accepted by the crew on L+3: one abdomen, one pelvis, and two chest radiographs; one chest aBMD radiograph, and one air aBMD exposure.

All images were immediately transmitted to an onboard computer and reviewed by the crew during the mission per protocol. Images were either rejected or accepted by the crew, and all accepted images were retained for analysis. Accepted in-flight radiographs are listed in Table 2. All radiographs were acquired by participating crewmembers without live ground support. Custom static visual and text guides supported step-by-step positioning and acquisition (Table S3).

**Table 2:** Radiographs Obtained Preflight, In-Flight, and Postflight

Group and No. of Examinations	Preflight	In-Flight	Postflight
Phantom	Phantom	Phantom (L+1), phantom plus watch (L+1)	Phantom
Non-crew operators	NA	NA	Hand, forearm, chest
Crew A (0 in-flight examinations and 10 total examinations)	Hand, forearm, chest*; aBMD: chest*; DXA: lumbar spine, femur, total spine	NA	DXA: lumbar spine, femur, total spine
Crew B (five in-flight examinations and 16 total examinations)	Hand, chest*, abdomen*, pelvis*; aBMD: chest; DXA: lumbar spine, femur, total spine	Hand (L+1)*, forearm (L+1)*, chest (L+3)*, abdomen (L+3)*, pelvis (L+3)*	DXA: lumbar spine, femur, total spine
Crew C (two in-flight examinations and 11 total examinations)	Hand, chest*; aBMD: chest*; DXA: lumbar spine, femur, total spine	Chest (L+3)*; aBMD: chest (L+3)*	DXA: lumbar spine, femur, total spine

Note.—All accepted in-flight radiographs are listed. Non-crew operators followed the same protocol as crewmembers. aBMD = areal bone mineral density, DXA = dual-energy x-ray absorptiometry, L+1 = day 1 after launch, L+3 = day 3 after launch, NA = not available.

* Scan scored by radiologists.

For stability during phantom imaging, hook and loop fasteners were used to secure the phantom to the detector. During anatomic imaging, the devices were handheld by crewmembers and occasionally stabilized against the spacecraft for support; no dedicated clamps or anchors were used. Radiographs were acquired with a source-image distance of 130 cm between the generator and detector, as measured with a tape measure.

Two radiation monitoring systems were used. First, passive dosimetry was used to monitor the in-flight radiation environment. Second, a Hybrid Electronic Radiation Assessor, or HERA, device was used to measure scattered radiation exposure from imaging.

### Postflight Protocol

After return to Earth, COTS hardware (x-ray generator, detector, and phantom) was examined for damage sustained during preflight qualification, in-flight operation, reentry, and recovery and was returned to the collaborating imaging companies. Standard phantom and anatomic radiographs were acquired using the in-flight protocol and hardware to evaluate x-ray generator performance. The x-ray detector was similarly visually inspected and underwent quality testing: Image analysis with and without x-rays was performed to assess detector behavior and image quality (eg, spatial resolution, contrast resolution, and electronic noise). An anthropomorphic chest phantom (Lungman; Kyoto Kagaku) was also imaged for qualitative assessment.

Postflight radiographs replicating preflight and in-flight investigations were part of the initial protocol. At the request of the crew and to minimize radiation exposure, only postflight dual-energy x-ray absorption scans were obtained (*n* = 3). The three crewmembers who used the digital radiography system also completed a survey 2 days after return (hereafter, R+2) that included Likert scale questions regarding ease of equipment use, the software graphical user interface, and examination protocol clarity. Open-ended questions regarding potential system improvements and broader interest in future spaceflight imaging were also included.

### Image Evaluation

Preflight, in-flight, and postflight data were tagged numerically, randomized, and anonymized to remove all private health information. Because the aBMD analysis approach is still being validated, those data and images were not included in this analysis.

The quality of preflight and in-flight radiographs acquired by the crew (*n* = 7 each) was rated by three independent radiologists: one with 10 years of experience and a fellowship in abdominal imaging; one with 6 years of experience and a fellowship in abdominal imaging; and one with 6 years of experience and a fellowship in musculoskeletal imaging. Raters were blinded to flight status and not otherwise involved in the study. Studies were read without use of consensus agreements. Picture archiving and communication system software was used to display the radiographic images; no autonomous interpretation was provided. The three raters viewed each radiograph independently on Barco Nio (MDNC-3421) monitors. They rated each radiograph on four image characteristics (overall image quality excluding positioning, spatial resolution, contrast resolution, and positioning) using a 5-point Likert scale, as follows: 1 = uninterpretable (nondiagnostic), 2 = poor (nondiagnostic), 3 = acceptable (diagnostic), 4 = good (diagnostic), and 5 = excellent (diagnostic).

Phantom and smartwatch radiographs were visually evaluated to assess spatial and contrast resolution for NDT. Crew survey responses regarding ease of use, graphical user interface, and protocol clarity were thematically analyzed to characterize crewmember perceptions, particularly with respect to future system improvements and priorities for future study. All survey questions and answer options presented to the radiologists and crewmembers are detailed in Appendix S1.

### Statistical Analysis

In all analyses, statistical significance was defined as α < .05; no correction was applied for multiple comparisons because this was a small feasibility study. Exact *P* values are reported. Image evaluation quality was assessed by calculating interrater reliability for the three image evaluators, using Gwet AC2 with quadratic weighting of discrepancies (23) as implemented in Python package irrCAC 4.0.4 (24). Due to excellent interrater reliability (AC2 = 0.97), subsequent analyses used the average of the three evaluators.

Preflight images were compared with in-flight images via nonparametric Wilcoxon rank sum tests as implemented in the “ranksum” function in Matlab version 24.2.0 (MathWorks), equivalent to the Mann-Whitney *U* test. Each of the four characteristics was compared separately. On a post hoc basis, the comparisons were repeated for central scans only (pelvis, abdomen, or chest; ie, excluding hand or forearm), which included seven of seven preflight images and five of seven in-flight images.

Multiple scans of the same participants were treated as independent because the study was designed to assess image quality rather than diagnostic performance. For crew survey responses (ease of use, graphical user interface, and protocol clarity), statistical analysis was not performed because of the small sample size (*n* = 3).

## Results

### Participant Characteristics

**Figure 1:**
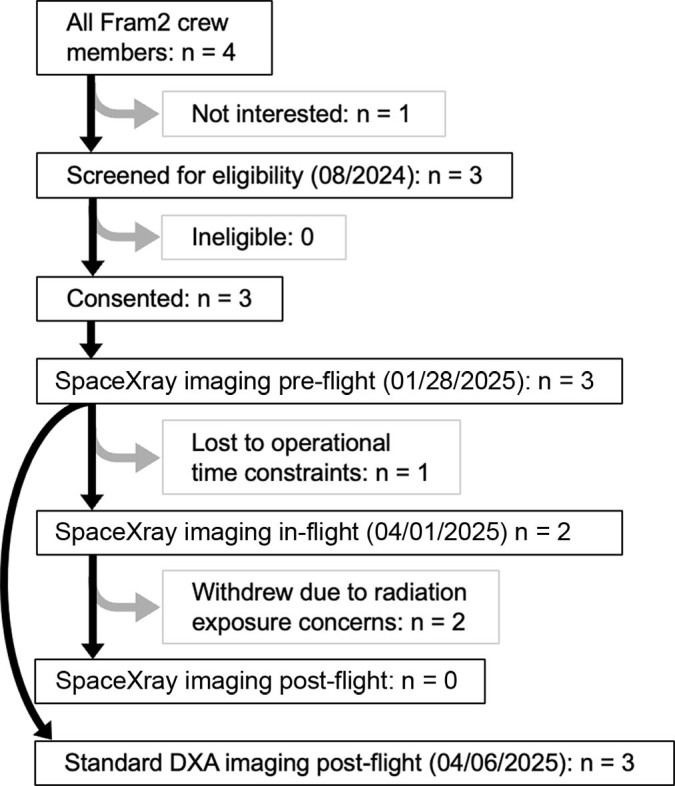
Flow diagram of participants. Date formats are MM/YYYY or MM/DD/YYYY. Three of four Fram2 astronauts participated. All three participants underwent preflight imaging. Only two underwent in-flight imaging due to operational time constraints on-orbit. Due to radiation exposure concerns, all crewmembers opted not to undergo postflight imaging with the SpaceXray system, although they consented to undergo re-imaging at Houston Methodist Hospital as part of the areal bone mineral density research protocol. Non-crewmembers underwent postflight imaging by the SpaceXray system following in-flight protocols to check hardware performance. DXA = dual x-ray absorptiometry.

As shown in Table 1, the crew (*n* = 4; two women) had a mean age of 43 years ± 14 (SD). Demographics of the three participating members are not provided separately because the small sample size could be potentially identifying; all potential participants were eligible and included. All three participants underwent preflight imaging at SpaceX and Houston Methodist Hospital, two underwent in-flight imaging due to on-orbit time constraints, and all three underwent postflight imaging at Houston Methodist Hospital (Fig 1).

### In-Flight Radiation Environment

All obtained radiographs are detailed in Table 2. Passive radiation dosimetry showed a total radiation dose of 1.5 mSv over the 3.5-day mission duration; this represents the combined radiation from the spaceflight environment and radiographic imaging and is approximately 10% higher than the daily radiation dose received by astronauts on the International Space Station. Depending on the cumulative number and types of radiographic investigation each crewmember received, the total estimated radiation exposure per participant from the SpaceXray system ranged from 0.3 mSv to 2.7 mSv (Table S4). Preliminary estimates of radiation exposure from scattered x-rays during imaging indicated that other crewmembers were exposed to approximately 0.0006 mSv on L+1 and 0.009 mSv ± 50% on L+3.

### In-Flight Nonmedical Imaging

**Figure 2:**
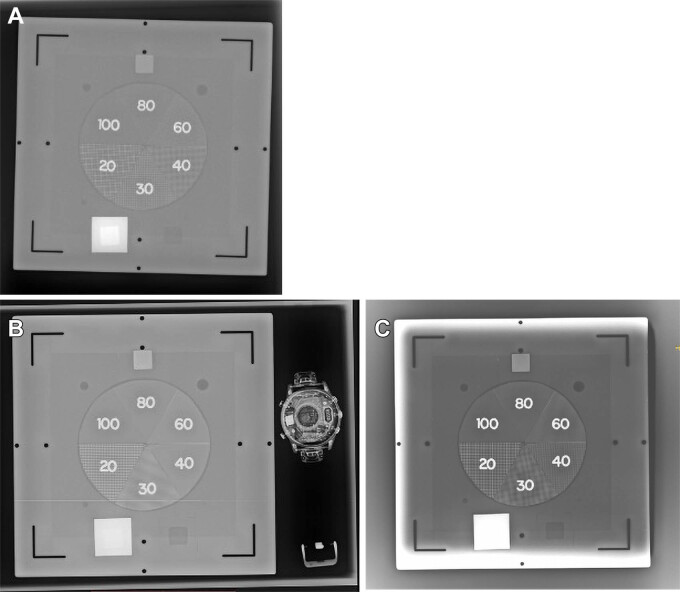
Preflight, in-flight, and postflight nonmedical radiographs. Radiographs of a quality control phantom were acquired **(A)** preflight by crew operators, **(B)** in-flight on day 1 after launch (L+1) by crew operators, and **(C)** postflight by a non-crew operator using the same imaging protocol. Numbers on the phantom represent the number of lines per inch to monitor resolution quality. Similar image quality was observed in preflight, in-flight, and postflight phantom radiographs.

Analysis of contrast resolution on in-flight phantom radiographs demonstrated that low-contrast targets were readily visible, including the smallest 2-mm target. High-contrast meshes ranging from 20 to 60 lines per inch were easily visualized, with 80 lines per inch remaining visible. As expected, 100 lines per inch were not visible, because this exceeds the Nyquist frequency of the detector (90.7 lines per inch). Similar image quality was observed in postflight phantom radiographs. Qualitative assessment of the in-flight smartwatch radiograph for NDT demonstrated clear visualization of internal components at the submillimeter scale (Fig 2).

### In-Flight Physiologic Imaging

**Figure 3:**
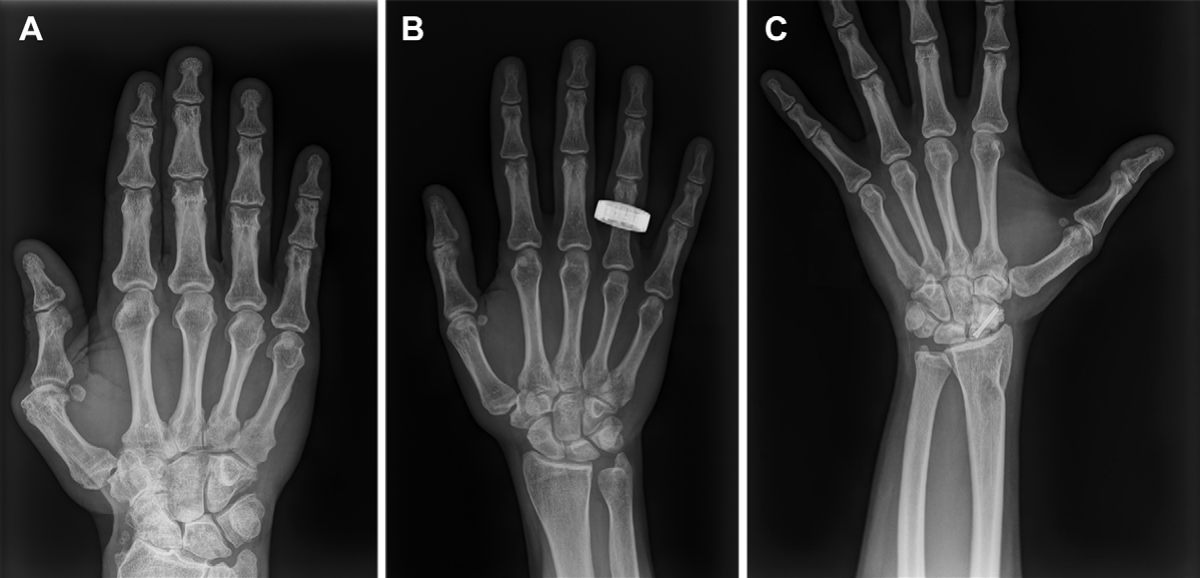
Representative preflight, in-flight, and postflight hand radiographs. Radiographs of the hand were acquired **(A)** preflight by a crewmember, **(B)** in-flight on day 1 after launch (L+1) by a crewmember, and **(C)** postflight by a non-crew operator using the same imaging protocol.

**Figure 4:**
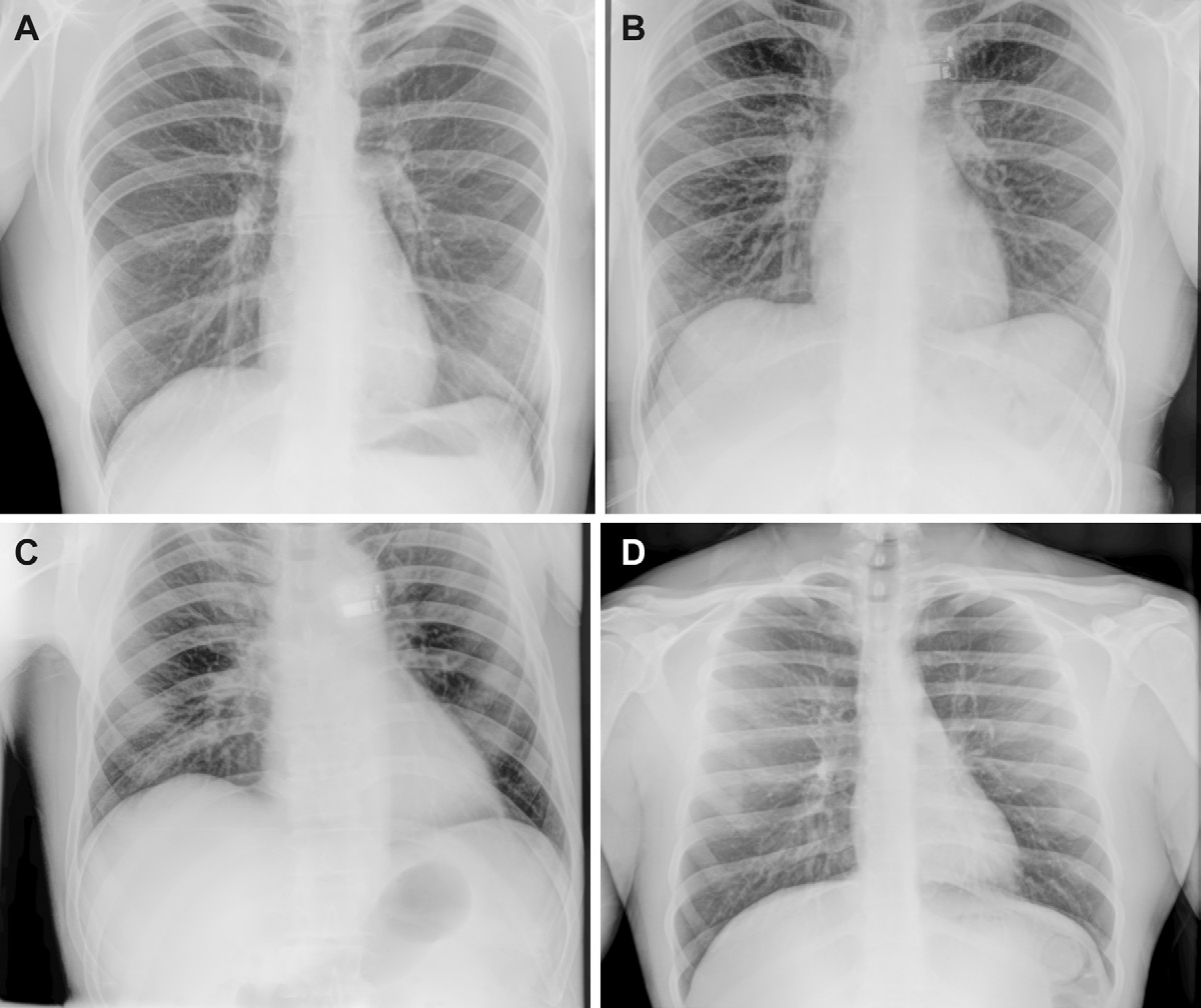
Representative preflight, in-flight, and postflight chest radiographs. Radiographs of the chest were acquired **(A)** preflight by a crewmember, **(B, C)** in-flight on day 3 after launch (L+3) by a crewmember, and **(D)** postflight by a non-crew operator using the same imaging protocol.

During imaging runs on L+1 and L+3, a total of seven images of crewmembers B and C were saved by the participants (Table 2). Representative preflight, in-flight, and postflight radiographs are shown in Figure 3 (hand) and Figure 4 (chest).

### Preflight Versus In-Flight Image Comparison

**Figure 5:**
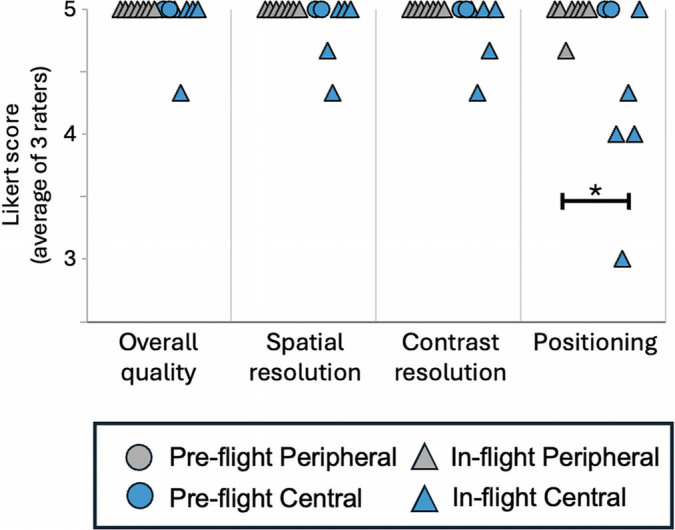
Graph shows comparison ratings for preflight and in-flight radiographs. Preflight and in-flight radiographs were acquired by crewmembers and independently evaluated on four characteristics: overall image quality, spatial resolution, contrast resolution, and positioning. A 5-point Likert scale was used, as follows: 1 = uninterpretable (nondiagnostic), 2 = poor (nondiagnostic), 3 = acceptable (diagnostic), 4 = good (diagnostic), and 5 = excellent (diagnostic). All radiographs scored greater than or equal to 3 for all characteristics, indicating diagnostic quality. Horizontal positioning of points within each category is for visual clarity. Comparing in-flight radiographs with preflight radiographs, no evidence of differences was found in overall image quality, contrast resolution, or spatial resolution, with a nonsignificant trend toward worse positioning in-flight. * = Wilcoxon rank sum *P* = .03 when comparing central radiographs (seven of seven preflight radiographs, five of seven in-flight radiographs).

Radiographs were assessed via qualitative evaluation of four characteristics: overall image quality (excluding positioning), contrast resolution, spatial resolution, and positioning. Independent radiologist evaluators provided a Likert score (range, 1–5) for each characteristic of each radiograph (Table S5). As interrater reliability was excellent (Gwet AC2 = 0.97), the raters’ scores were averaged to produce a single score for each radiograph and characteristic (Fig 5).

Comparing in-flight radiographs with preflight radiographs, we found no evidence of differences in overall image quality, contrast resolution, or spatial resolution, with a nonsignificant trend toward worse positioning in-flight (Table 3, top). Notably, this trend toward worse in-flight positioning was driven by low in-flight positioning scores for central radiographs (mean score for chest, pelvis, and abdomen, 4.07 ± 0.72) but not peripheral radiographs (mean score for arm and hand, 5 ± 0), as shown in Figure 5 (triangles vs circles).

**Table 3:** Preflight Versus In-Flight Image Comparison

Characteristic	Preflight Mean	Preflight SD	In-Flight Mean	In-Flight SD	*P* Value	W Statistic
All radiographs	7		7			
Overall quality	5.00	0	4.90	0.25	>.99	59.5
Contrast resolution	5.00	0	4.85	0.26	.46	59.5
Spatial resolution	5.00	0	4.85	0.26	.46	56.0
Positioning	4.95	0.13	4.33	0.75	.07	65.0
Central radiographs	7		5			
Overall quality	5.00	0	4.87	0.30	.83	49.0
Contrast resolution	5.00	0	4.80	0.30	.30	52.5
Spatial resolution	5.00	0	4.80	0.30	.30	52.5
Positioning	4.95	0.13	4.07	0.72	.02*	59.0

Note.—Unless otherwise specified, values are Likert scores (range, 1–5). Descriptive statistics are means and SDs across radiographs. Statistics are comparisons between preflight and in-flight images via the Wilcoxon rank sum test.

* *P* < .05.

To better quantify preflight versus in-flight differences for radiographs of central targets only, we repeated the analysis for central targets (seven of seven preflight radiographs, five of seven in-flight radiographs). As shown in Table 3, bottom, we again found no evidence of differences between in-flight radiographs and preflight radiographs in overall image quality, contrast resolution, or spatial resolution; however, for central body radiographs, positioning was significantly worse in-flight than preflight.

Overall image quality metric achieved a diagnostic level (Likert score ≥3) for all radiographs, with all scores greater than or equal to 4 for all raters and radiographs, despite the worse in-flight positioning of central (chest, pelvis, and abdomen) radiographs.

### Post-Flight Crew Survey

**Figure 6:**
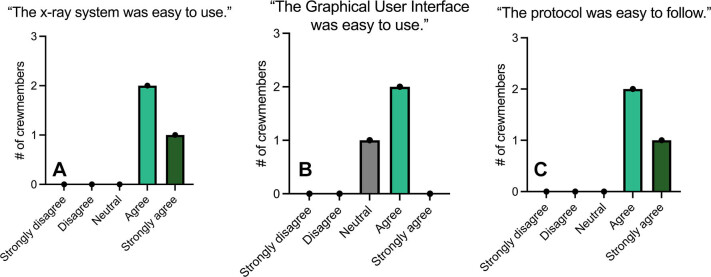
Charts show crewmember survey results regarding the operational use of the x-ray system (*n* = 3, 2 days after return [R+2]). All three crewmembers who used the system completed a survey at R+2 that included a five-option Likert scale: “strongly agree,” “agree,” “neutral,” “disagree,” and “strongly disagree.” The three questions were as follows: **(A)** “The x-ray system was easy to operate,” **(B)** “The Graphical User Interface was easy to use,” and **(C)** “The protocol was easy to follow.” The full survey questions and answer options are provided in Appendix S1.

The three crewmembers who used the digital radiography system completed a survey on R+2 regarding ease of equipment operation (physical operation of the x-ray generator), the software graphical user interface, and examination protocol clarity (Fig 6). All crewmembers agreed (*n* = 2) or strongly agreed (*n* = 1) that the x-ray system was easy to use and the protocol was easy to follow. Most crewmembers (*n* = 2) rated the graphical user interface as easy to use, while one crewmember was neutral.

Crewmembers were also asked open-ended questions regarding potential system improvements, protocol changes, and additional imaging modalities for future implementation (Table 4). All respondents noted a need for improved mechanisms to securely mount and clamp both the x-ray detector and generator. Likely secondary to operational challenges and time constraints while on-orbit, one crewmember reported imaging only the hand and forearm and expressed interest in being able to image the chest. Crewmembers also expressed interest in imaging additional body regions, including distal joints, and in incorporating other imaging modalities, such as MRI.

**Table 4:** Crewmember Survey Results Regarding Future Implementation of Portable Imaging for Spaceflight Applications

Question	Collated Answers
Suggestions for improved ease of operation	Equipment:
	• “Dedicated clamps to secure both the generator and detector.”
	• “More hook Velcro to secure generator and detector…clamps would work for mounting the generator and detector securely.”
	• “Velcro on the X-ray detector and generator.”
	User interface:
	• “Label STEP 1; STEP 2 etc to easily guide the process.”
Interest in additional images	• “Knee joint.”
	• “Chest. I only imaged my had [sic] and forearm.”
	• “Ankle and foot also would be very interesting. And femur.”
Interest in additional imaging modalities	• “MRI would be interesting”
	• “CT scan”
	• “Sure! We had an ultrasound but MRI would be interesting.”

Note.—Survey results were collected from three crewmembers 2 days after return from orbit. All participants noted the need for improved mechanisms to secure the detector and generator panels. Crewmembers also expressed substantial interest in additional imaging; one reported being unable to obtain their own chest radiograph, likely due to operational time constraints during the mission. Multiple crewmembers further indicated interest in expanding imaging modalities for future spaceflight applications, including MRI or CT scans.

### Post-Flight Hardware Evaluation

Postflight performance of the x-ray generator and detector appeared unchanged on manufacturer assessment. Superficial structural damage was sustained by the x-ray generator during landing and recovery, including bent skin guards and a broken plastic lens on the collimator (Fig S1); these did not affect internal hardware components or x-ray output. Similarly, the detector passed visual inspection and quality control tests, demonstrating acceptable spatial resolution and electronic noise.

Postflight radiographs acquired by non-crewmembers following the same in-flight protocols showed no qualitative differences compared with preflight or in-flight radiographs. Radiographs of an anthropomorphic chest phantom were qualitatively assessed as normal and comparable to preflight performance.

## Discussion

Digital radiography has the potential to expand in-flight imaging during spaceflight, improving diagnostic quality over US alone while enabling nondestructive testing. The key aim of this study was to establish the feasibility of a portable, digital, commercial-off-the-shelf radiography system for on-orbit anatomic and equipment imaging applications. The SpaceXray system enabled a nonmedical crew with 4 hours of preflight training to acquire radiographic images during the Fram2 orbital flight. While central body images (chest, pelvis, and abdomen) had worse positioning in-flight, in-flight radiographs were equivalent to preflight radiographs in overall image quality, spatial resolution, and contrast resolution.

Crew survey results reflected these operational challenges with subject positioning and alignment—yet despite the challenges of subject positioning in microgravity, in all cases the SpaceXray system acquired diagnostically adequate radiographs. Equipment and phantom tests of in-flight radiographs showed high spatial resolution, despite increased ambient radiation during Fram2’s polar orbital flight compared with nonpolar inclinations (25–28). In-flight radiographs achieved contrast resolution up to 80 lines per inch, and in-flight NDT radiographs clearly visualized submillimeter internal equipment components. Successful NDT may have important applications for monitoring spacesuit damage, as spacesuits contain critical internal components that cannot be disassembled for inspection.

Estimated radiation exposure to participants from the x-ray system was not greater than that associated with standard clinical imaging on Earth (0.3 mSv–2.7 mSv). Additional radiation exposure to other crewmembers and the spacecraft was minimal (0.009 mSv ± 50%). Differences in scattered-radiation exposure between L+1 (0.0006 mSv) and L+3 (0.008 mSv) may reflect differences in x-ray generator output settings, the number of investigations performed, or generator orientations during imaging sessions. These low radiation exposures suggest that in-flight monitoring of applications, such as assessment of bone loss using quantitative aBMD from preflight and in-flight radiographs, may be relatively low risk (18,29,30). Similarly, the clinical use of in-flight radiography, either as a standalone modality or as a complement to portable US, is unlikely to increase medical risks beyond accepted terrestrial radiation-exposure guidelines (31–36). However, prospective studies will be required to establish guidelines for in-flight examination indications, image interpretation, and imaging baselines—particularly for gravity-dependent findings such as bowel gas patterns.

Provision of care in terrestrial settings may also benefit from advances in radiographic system ruggedization, portability, and image-analysis tools. Potential applications range from medical evacuations in austere environments (eg, combat zones) to infectious disease screening in remote, resource-limited communities, including expanded tuberculosis screening (9,37). Ultimately, communities both on and off Earth may benefit from advances in ultraportable digital radiographic imaging systems.

Future work should specialize the COTS system for spaceflight. Ruggedization of the radiography system through vacuum hardening may further enable NDT for external spacecraft structural surveillance (38) and may also reduce superficial damage caused during descent operations, although postflight system performance was unaffected. In addition, development of embedded support tools and artificial intelligence–based assistance may help crews identify radiographs with inadequate positioning (9,10). In addition, future work should refine examination protocols, develop spacecraft-agnostic securement mechanisms, and implement rapid autonomous image-interpretation tools. Finally, additional studies are needed to establish protocols for imaging acutely ill, incapacitated, or immobilized crewmembers.

This study had four major limitations. First, limited in-flight imaging time restricted the number and variety of radiographic images obtained, opportunities to correct positioning with repeated exposures, and the number of crewmembers who could participate. Limited preflight training and absence of teleguidance were operationally realistic; nonetheless, diagnostically adequate images were obtained for most examination types. Second, radiographs were reviewed on Earth, but real-time telehealth may be unfeasible during exploration-class missions to the Moon or Mars. Third, the small sample size and inclusion of only healthy, able-bodied crewmembers limit generalizability to clinical scenarios involving pain or agitation. Fourth, NDT was limited to hardware that could be positioned between the x-ray generator and detector, precluding assessment of large equipment, external spacecraft structures, or large-scale habitat surveys.

In conclusion, this study demonstrated the first on-orbit acquisition of diagnostically adequate human radiographs. The user-friendly design of our commercial-off-the-shelf (COTS) system enabled rapid performance of numerous imaging examinations despite limited in-flight operational time, minimal crew training, and the absence of anchoring equipment in the microgravity environment. Both human anatomic imaging and equipment imaging for nondestructive testing were carried out in-flight without loss of image quality. Further miniaturization, ruggedization, and improved usability of this COTS radiography system may enable its eventual inclusion in future mission medical kits as operational constraints allow.

## Supplemental Files

Appendix S1, Tables S1-S5, Figure S1

Conflicts of Interest
