# Supplementary material for: SpaceXray: Feasibility and Diagnostic Capabilities of On-Orbit Medical Radiography
Source: Radiology. 2026 Jul 14;320(1):e260258. doi: 10.1148/radiol.260258 (PMC13421204; doi:10.1148/radiol.260258)
Supplement: Appendix S1, Tables S1-S5, Figure S1 [file ry260258suppa1.pdf]

Supplemental materials undergo peer review but are not copyedited, and may include typographical errors.

©RSNA, 2026
10.1148/radiol.260258

SpaceXray: Feasibility and Diagnostic Capabilities of On-Orbit Medical Radiography

Appendix S1: Supplementary Information

Table of Contents:

Figure S1: Images of the portable x-ray system used	2
Portable X-Ray Generator Technical Specifications	3
Portable X-Ray Detector Technical Specifications	7
Table S1: Key technical specifications of the MinXray TR90BH portable X-ray generator	8
Table S2: Key technical specifications of the KA Revel portable X-ray receiver panel	9
Table S3: Positioning Protocols	10
Table S4: Dosimetry estimates from X-ray imaging in standard clinical practice and during Fram2	11
Table S5: Reviewer scores for all rated X-ray images	12
Qualitative Radiographic Assessment Questions	13
Crew Survey Questions	14

Supplemental materials undergo peer review but are not copyedited, and may include typographical errors.

Figure S1: Images of the portable x-ray generator (**A**) and detector (**B**) used in this study. **C**) Shows the minor bent skin guards on the X-ray generator that were found post-flight.

A) X-ray generator

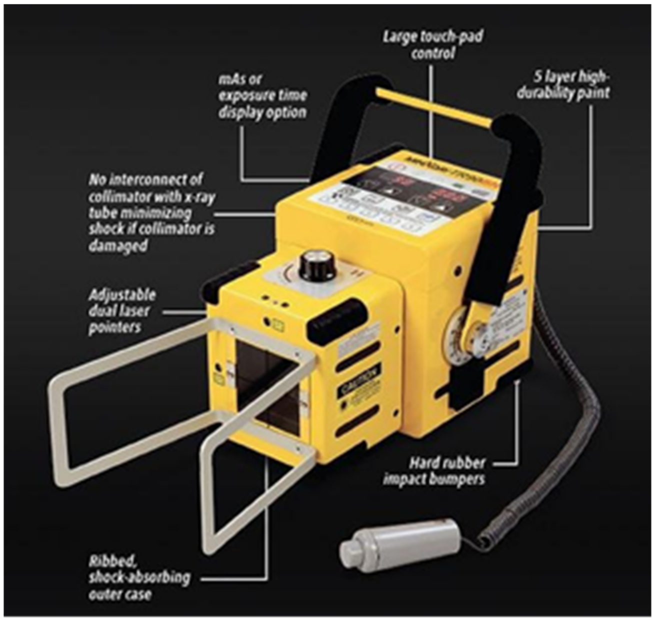

B) X-ray detector

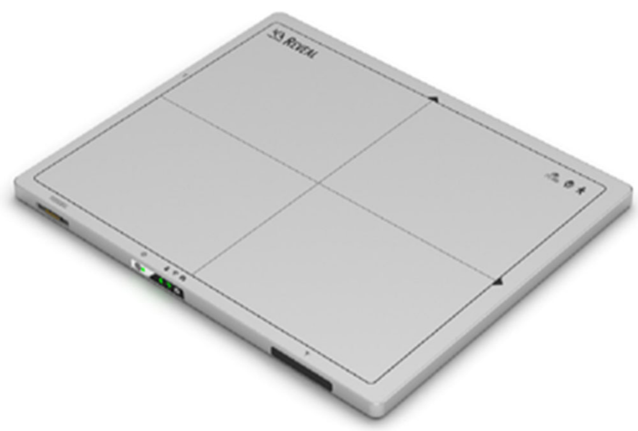

C) X-ray generator post-flight

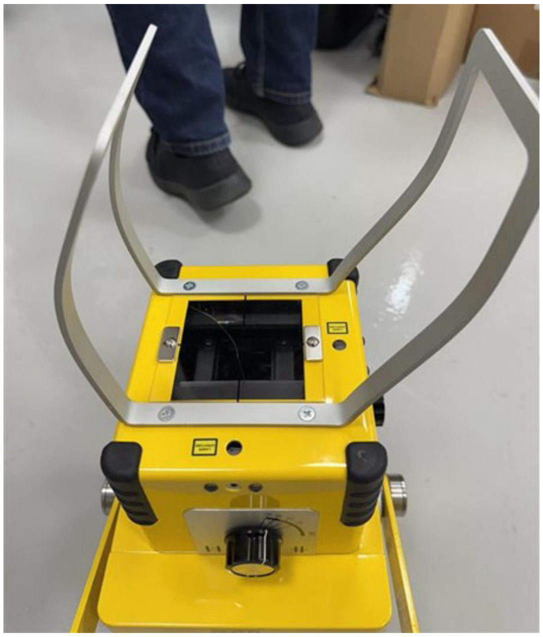

Supplemental materials undergo peer review but are not copyedited, and may include typographical errors.

Technical Specifications of the MinXray TR90BH Portable X-ray Unit

The MinXray TR90BH is a self-contained, battery-powered portable X-ray generator designed for high-frequency X-ray output in field settings. X-rays are produced by a tungsten-anode X-ray tube energized via an internal high-voltage inverter, with all necessary electronics (exposure timing, kV/mA control, and safety interlocks) integrated into the compact unit. **Table S1** summarizes the key performance specifications of the TR90BH.

High-Voltage Inverter and Control Electronics

At the core of the TR90BH is a high-frequency inverter that converts the battery's low-voltage DC output into the high-voltage DC needed to drive the X-ray tube. The inverter operates at up to ~150 kHz in a resonant mode, using high-voltage transformer circuits to step 57.6 V up to the selected tube potential (40–90 kV DC). A microprocessor-based control board (MinXray part MT5002A) regulates this inverter and governs all exposure settings. The tube voltage is selectable in 2 kV steps via the user interface and maintained within ±10% accuracy by feedback control. Calibration potentiometers on the MT5002A board (e.g. VR2 for 40–60 kV and VR3 for 62–90 kV ranges) allow fine adjustment of the peak kV output. This board also contains the exposure timing circuitry: a rotary switch (SW1) on the MT5002A sets the base exposure duration, with each switch step adding a fixed increment (~5–13 ms) to all timer settings for calibration. Exposure times from 0.02 s up to 1.0 s are available in normal mode (with a ±10% or ±1 ms tolerance). An internal exposure timer IC or microcontroller triggers the high-voltage generation for the selected duration and also enforces the maximum exposure limit of ~0.3 s in the High-Power mode.

A second circuit board, MT1002A, interfaces with the high-voltage section and the X-ray tube. This board includes the filament driver and monitoring circuitry for tube current (mA) and tube voltage (kV). When an exposure is initiated, the control electronics first activate the filament heating circuit to bring the tube's cathode filament up to operating temperature (indicated by a "ready" signal once the tube is thermionically emission-ready). The TR90BH uses a two-stage exposure switch: pressing the first stage powers the filament (and enables the "READY" indicator), and the second stage applies the high-voltage to generate X-rays. The filament current is regulated such that the desired tube current will flow when the high voltage is applied; the system uses feedback from an internal current-sense circuit (with test points TP5 and TP6 on the MT1002A board for kV and mA, respectively) to monitor the actual tube voltage and current during exposure. If needed, service technicians can adjust trim potentiometers on the control board (VR5, VR6 on MT5002A) to calibrate the tube current output across the kV range, ensuring that, for example, at 90 kV the tube current does not exceed ~10 mA in normal mode or ~15 mA in High-Power mode. The high-voltage generation is thus a closed-loop system: the control board modulates the inverter drive and filament current in real time to maintain the set kV and mA, and it terminates the exposure after the programmed time or if any safety threshold is reached.

X-ray Tube and Emission Characteristics

X-rays are generated in a dedicated dual-focus X-ray tube (model D-0814M, Canon Electron Tubes & Devices) with a tungsten anode target. The tube features a 0.8 mm focal spot (per IEC 336) and a target angle of 16°, typical for general-purpose radiography. It is a stationary anode tube with an anode heat storage capacity of about 9.86 kHU (≈7 kJ). The high-voltage inverter

Supplemental materials undergo peer review but are not copyedited, and may include typographical errors.

applies a constant potential (CP) high voltage across the tube during each exposure, accelerating electrons from the heated cathode filament to the anode target. The tube current – which corresponds to the electron beam intensity – is adjustable via filament temperature and is automatically limited by the control system depending on the selected kV. At lower tube voltages (40–60 kV) the TR90BH can drive up to 20 mA of tube current, while at the maximum 90 kV setting the standard tube current is about 10 mA. This trade-off prevents excessive load on the high-voltage supply and tube; however, a High Power (HP) mode can be engaged to boost the tube current to ~15 mA even at 82–90 kV, effectively yielding a ~50% higher X-ray output in that range. The HP mode is limited to short exposures (≤0.3 s) to avoid overheating the anode overtaxing the battery. An anode cooling period is inherently enforced by the duty cycle (discussed below), and the unit's firmware will error out or prevent re-exposure if tube temperature or current draw exceeds safe limits (e.g., an error code is triggered for over-current at high kV when the battery is low). To ensure stable X-ray output and tube longevity, the TR90BH employs warm-up protocols for the X-ray tube. At the beginning of each use (e.g., each day or after long idle periods), the manufacturer prescribes a tube seasoning routine: a series of low to high kV exposures with increasing energy. For example, a typical daily warm-up is to take one exposure at 40 kV/0.2 s, one at 70 kV/0.2 s, and one at 90 kV/0.2 s, each spaced by ~12 seconds. If the unit has been idle for over a month, a more extensive seasoning is recommended (e.g. two exposures at each of 40 kV, 70 kV, 90 kV). This process helps remove any gas buildup in the tube and prevents arcing or abnormal discharges in the tube by gradually restoring the vacuum integrity. The TR90BH's control circuit will inhibit operation or shut down if abnormal tube voltage discharge is detected, as an additional protective measure. After proper warm-up, the tube can reliably produce X-rays at the full range of technique factors listed in Table 1.

The X-ray beam is collimated and filtered for safety and image quality. The tube assembly provides an inherent filtration equivalent to at least 0.8 mm Al from the glass envelope and oilbath, and additional fixed filters in the beam limiting device bring the total inherent filtration to approximately 2.0 mm Al equivalence. The TR90BH's integrated light-beam collimator (Mikasa R-300V) adds further filtration (e.g. through its mirror and aperture windows), for a guaranteed ≥3.0 mm Al total filtration of the X-ray beam. This filtration removes low-energy "soft" X-rays from the beam that would otherwise increase patient skin dose without contributing to image formation. The collimator includes lead shutters to restrict the beam to the region of interest, dual integrated laser pointers for positioning (class IIIa, <3 mW @ 635 nm), and a light field lamp for aligning the beam. These features help minimize unnecessary radiation and ensure accurate aiming, which is especially important given the unit's use in varied environments (including potentially confined research setups). The TR90BH also has visual and audible X-ray indicators that activate during X-ray emission as mandated by safety standards, alerting the operator and bystanders when radiation is being produced.

Battery Power System and Exposure Operation

The TR90BH is powered by a high-capacity rechargeable lithium-ion battery (MinXray M910BL pack). This battery is a 57.6 V DC pack (nominal, 16-cell series arrangement in four 14.4 V modules), with a capacity of 1.9 Ah (~110 Wh). It is capable of delivering up to 40 A continuous current to drive the X-ray inverter during high-power exposures. The battery pack is physically small (160×80×62 mm, ~1.1 kg) and is designed for quick swapping in the field. A battery level indicator on the unit (with a four-LED bar display) provides feedback on the state of charge, and the system will warn or prevent operation if the battery is critically low (to avoid a

Supplemental materials undergo peer review but are not copyedited, and may include typographical errors.

mid-exposure power drop). Under typical usage, one full charge can yield on the order of several hundred exposures (300–500, depending on technique factors) before depletion. The battery is recharged with an included multi-stage charger (MinXray M120BC): the charger uses a constant-current (~3.2 A) charge phase followed by a constant-voltage hold at 16.8 V per module (approximately 67.2 V total across the four-series pack when balanced). A full recharge cycle takes about 3–4 hours to complete. The charging procedure involves removing the battery from the X-ray unit and connecting it to the charger dock, which is powered by an AC adapter (90–240 V AC input). The charger's built-in circuitry ensures each 14.4 V module is balanced and prevents over-charge; indicator LEDs signify charging status and completion. For longevity, the battery is recommended to be stored with a partial charge (~50%) if not used for extended periods, and it should be recharged at least every 5 months to avoid deep discharge damage.

In operation, the TR90BH's battery provides a very stable DC supply for the inverter, enabling constant potential X-ray output similar to line-powered units. The absence of cable tethering and the high output (up to 90 kV, 15 mA) make the unit suitable for radiographic imaging in remote or microgravity environments where standard X-ray equipment is impractical. The control electronics manage the battery draw to maintain output: at maximum technique (90 kV, 15 mA) the inverter will pull about 40 A from the battery. As a safety measure, the system monitors battery voltage sag and will abort or prevent exposures if the battery cannot sustain the required voltage (an error code P03 indicates the battery was too low to support a high-load exposure). The unit is internally powered (Battery Operated, Class II), meaning it has no mains connection during use, which provides isolation from electrical shock hazards. For additional safety, when the device is not in use for long periods, the battery should be removed from the unit to eliminate any parasitic drain on the cells by the control circuitry.

Operating Modes, Duty Cycle and Safety Considerations

Because of its compact design, the TR90BH is rated for intermittent use. The specified duty cycle is 1:60, meaning for every 1 second of X-ray exposure, a 60-second cool-down is required. In practice, this is illustrated as one 0.2 s exposure every 12 seconds (which is 1:60) or, equivalently, one 1.0 s exposure per 60 s interval. This duty cycle ensures that the X-ray tube anode and the high-voltage inverter do not overheat and that the battery is not over-stressed. The control system keeps track of recent exposures and will enforce this limit; if an operator attempts exposures in excess of the duty cycle, the unit may delay the next exposure or shut down until a safe interval has passed. In High-Power mode, because each exposure deposits more heat, adhering to the duty cycle is even more critical (e.g. a full 0.3 s high-power exposure should be followed by a proportionally longer rest). The anode heat capacity (7 kJ) allows for a few high technique shots in short succession, but prolonged use at high settings will approach thermal limits. The tube housing is filled with insulating oil that helps dissipate heat from the anode into the housing. There are no actively powered cooling fans in the unit – cooling is purely passive via the tube housing and casing. Therefore, the intermittent operation specification must be followed to avoid activating thermal cutouts. If the internal temperature sensors detect an unsafe rise (or if an "unusual discharge" from the tube is sensed, possibly due to overheating or arcing), the TR90BH will initiate an automatic safety shutdown. Recovery typically requires a few minutes of cooling and, if applicable, a power-cycle of the unit.

The TR90BH incorporates multiple radiation safety features to protect the user and subjects. As mentioned, a two-stage deadman exposure switch is used, ensuring that X-rays are only generated when the operator intentionally presses and holds the expose button through the

Supplemental materials undergo peer review but are not copyedited, and may include typographical errors.

"prep" and "expose" stages. An X-ray ON indicator light (and audible buzzer) alerts when X-rays are being emitted, and a "READY" indicator confirms the device is prepared for exposure (filament warmed and all systems nominal). The beam limiting device not only confines the beam to the desired area but also has shielded enclosure walls to reduce leakage radiation. The TR90BH's design complies with international standards for leakage radiation: the leakage is kept below the regulatory limit (e.g., <0.88 mGy/hr at 1 m at full output, per FDA/IEC requirements) by lead shielding in the tube assembly. The unit's housing and internal structures are likewise shielded or grounded appropriately to contain high voltage and prevent electrical shock to users. All user-accessible external surfaces of the X-ray head remain at safe temperatures and potentials during operation, consistent with it being a Type BF applied part in medical electrical equipment classification (no risk of shock from the patient-side).

The MinXray TR90BH generates X-rays through a combination of a high-frequency DC inverter and a robust fixed-anode X-ray tube, all orchestrated by sophisticated internal electronics to achieve precise control of exposure parameters. Its battery-powered design and High-Power mode provide significant flexibility and output for off-grid radiography, while built-in timers, interlocks, and thermal/radiation safeguards ensure that the X-ray generation is both reliable and safe under the operating conditions required for microgravity research applications. The unit's internal boards (MT5002A control board and MT1002A high-voltage board) coordinate to maintain the selected kV and mA during the exposure, and its power system and cooling constraints are managed via firmware to prevent overload. As a result, the TR90BH can deliver consistent X-ray output (up to 90 kV, 15 mA) in a portable form factor, making it well suited for use in remote environments or specialized setups. Design elements work in concert to enable the safe generation of X-rays without compromising on performance or safety.

Supplemental materials undergo peer review but are not copyedited, and may include typographical errors.

Technical Specifications of the Reveal™ 35C Flat Panel Detector

The Reveal™ 35C (KA Imaging Inc., Waterloo, ON, Canada) is a 14 × 17 inch (35 × 43 cm; ISO 4090 compliant) flat panel digital radiography detector. The detector has a pixel pitch of 140 µm and a total weight of 3.6 kg (8 lbs), including the battery. The sensing layer consists of amorphous silicon coupled with a cesium iodide (CsI) scintillator. The system supports both wired and wireless data transmission. Wired communication is via Gigabit Ethernet (1000BASE-T), and wireless communication is supported through IEEE 802.11 ac/n standards. Image acquisition is triggered using a lossless automatic exposure detection (AED) mechanism.

Power and Electrical Specifications

The detector operates using hot-swappable batteries and/or tethered power. When connected to AC power, it supports an input range of 100–240 VAC at 50–60 Hz. Analog-to-digital (A/D) conversion is performed at 16-bit resolution.

Performance Characteristics

The typical imaging cycle time is approximately 20 seconds, with a typical preview time of 7 seconds. The system operates over an energy range of 40–150 keV.

Dose Efficiency and Spatial Resolution

Dose efficiency and resolution performance were measured under RQA5 beam quality conditions. The detector's detective quantum efficiency (DQE) and modulation transfer function (MTF) at selected spatial frequencies are summarized in **Table S2**. The saturation dose is specified at 140 µGy, and the maximum linear dose is 120 µGy. Dose efficiency and resolution values were measured under RQA5 conditions.

Supplemental materials undergo peer review but are not copyedited, and may include typographical errors.

Table S1: *Key technical specifications of the MinXray TR90BH portable X-ray generator*

Parameter	Specification
Tube voltage range	40–90 kV (DC), in 2 kV increments (±10% accuracy)
Tube current (normal mode)	20 mA @ 40–60 kV; 15 mA @ 62–80 kV; 10 mA @ 82–90 kV (±20%)
Tube current (High-Power)	15 mA @ 82–90 kV (High-Power mode, 50% boost at high kV)
Exposure time range	0.02 – 1.0 s (±10% +1 ms); up to 0.3 s max in High-Power mode
X-ray output power	Up to 1.35 kW (e.g., 90 kV @ 15 mA in High-Power mode)
X-ray tube	Canon/Toshiba D-0814M, 0.8 mm focal spot, 16° W anode
Anode heat capacity	~9.86 kHU (7 kJ) (stationary anode)
Inherent filtration	≥2.0 mm Al (incl. 0.8 mm from tube insert)
Total filtration	≥3.0 mm Al (with integrated collimator)
Power source	Detachable 57.6 V Li-ion battery pack (1.9 Ah, 110 Wh)
Battery capacity	~300–500 exposures per charge (typical use)
Battery recharge	3–4 hours (via 19 V AC adaptor and CC/CV charger)
Duty cycle	1:60 (e.g., one 0.2 s exposure per 12 s)<br>(continuous operation with intermittent loading)

Supplemental materials undergo peer review but are not copyedited, and may include typographical errors.

Table S2. *Key technical specifications of the KA Imaging Reveal 35C Flat Panel Detector. All values measured under RQA5 beam quality conditions. DQE = Detective Quantum Efficiency; MTF = Modulation Transfer Function.*

Spatial Frequency (lp/mm)	DQE (%)	MTF (%)
0	75	N/A
1	67	64
2	53	35
3	34	18
Nyquist Frequency	21	13

Supplemental materials undergo peer review but are not copyedited, and may include typographical errors.

Table S3*: Positioning protocols similar to these were used in the SpaceXray research protocol to guide the crew on orbit.*

**Anatomy**	**Alignment Guidance**	**Anticipated Image**
**Hand**	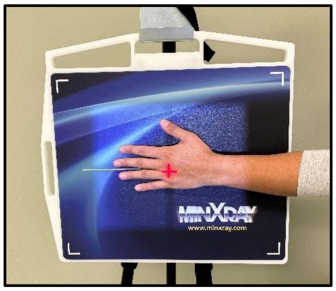	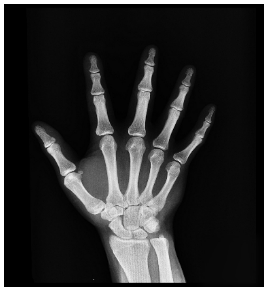
**Forearm**	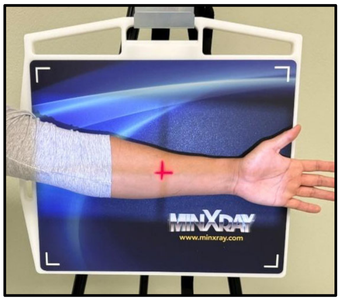	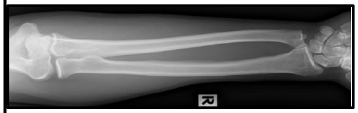
**Chest**	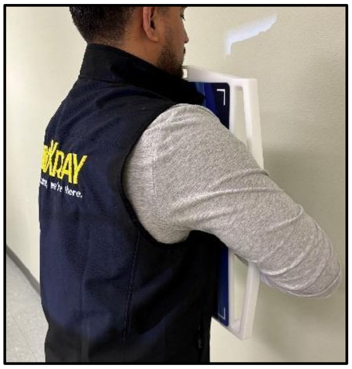	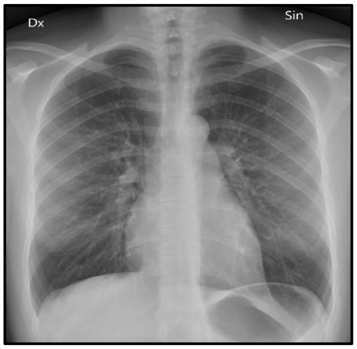

Supplemental materials undergo peer review but are not copyedited, and may include typographical errors.

Table S4*: Dosimetry estimates from X-ray imaging in standard clinical practice and during Fram2.* *Dose estimates from general clinical practice on Earth for various radiographic X-ray imaging are provided. They are compared with dosimetry estimates of ionizing radiation exposure provided by the manufacturer at 130 cm source-image distance (SID) for the same imaging exams. Using these dose estimates for each exam, the total exposure each crewmember received from the SpaceXray system was calculated and ranged from 0.3 mSv - 2.67 mSv of dose equivalent radiation.*

**Exam**	**Dose Estimates in Standard Clinical Practice**	**Dose Estimates for Fram2 by the X-Ray Generator Manufacturers**			
	Dose Estimate (mSv)	*kV*	*mA*	*Radiation Exposure (mR)*	*Dose Estimate (mSv)*
Hand	0.0005	54	3.2	2.7432	0.03
Forearm	0.001	54	3.2	2.7432	0.03
Abdomen	0.5	84	10	35.82	0.36
Pelvis	0.7	84	10	35.82	0.36
Chest	0.02	90	2.8	11.96	0.12
Chest (aBMD)	0.02	90	2.8	11.96	0.12

Supplemental materials undergo peer review but are not copyedited, and may include typographical errors.

Table S5: *Pre-flight and in-flight image scores*

** Taken while following an aBMD protocol*

	Exam	Spatial Resolution			Contrast Resolution			Overall Quality			Positioning		
		# 1	#2	# 3	# 1	# 2	# 3	# 1	# 2	# 3	# 1	# 2	# 3
Pre-flight	1 - Pelvis	5	5	5	5	5	5	5	5	5	5	4	5
	2 - Abdomen	5	5	5	5	5	5	5	5	5	5	5	5
	3 - Chest	5	5	5	5	5	5	5	5	5	5	5	5
	4 - Chest	5	5	5	5	5	5	5	5	5	5	5	5
	5 - Chest*	5	5	5	5	5	5	5	5	5	5	5	5
	6 - Chest	5	5	5	5	5	5	5	5	5	5	5	5
	7 - Chest*	5	5	5	5	5	5	5	5	5	5	5	5
In-flight	1 - Hand	5	5	5	5	5	5	5	5	5	5	5	5
	2 - Forearm	5	5	5	5	5	5	5	5	5	5	5	5
	3 - Chest	5	5	4	5	5	5	5	5	5	5	3	4
	4 - Pelvis	5	4	4	5	4	4	5	4	4	3	2	4
	5 - Abdomen	5	5	5	5	5	5	5	5	5	4	5	4
	6 - Chest	5	5	5	5	5	4	5	5	5	3	5	4
	7 - Chest*	5	5	5	5	5	5	5	5	5	5	5	5

Supplemental materials undergo peer review but are not copyedited, and may include typographical errors.

Qualitative Radiographic Assessment Questions

A team of licensed radiologists were provided with pre-flight and in-flight X-rays and blinded to the timepoint for the image. They were asked to review each image using the following assessment:

1. Rate the image on a scale of 1-5 for its overall image quality (outside of positioning).
   1 - uninterpretable (non-diagnostic)
   2 - poor (non-diagnostic)
   3 - acceptable (diagnostic)
   4 - good (diagnostic)
   5 - excellent (diagnostic)

2. Rate the image on a scale of 1-5 for its spatial resolution.
   1 - uninterpretable (non-diagnostic)
   2 - poor (non-diagnostic)
   3 - acceptable (diagnostic)
   4 - good (diagnostic)
   5 - excellent (diagnostic)

3. Rate the image on a scale of 1-5 for its contrast resolution.
   1 - uninterpretable (non-diagnostic)
   2 - poor (non-diagnostic)
   3 - acceptable (diagnostic)
   4 - good (diagnostic)
   5 - excellent (diagnostic)

4. Rate the image on a scale of 1-5 for its positioning.
   1 - uninterpretable (non-diagnostic)
   2 - poor (non-diagnostic)
   3 - acceptable (diagnostic)
   4 - good (diagnostic)
   5 - excellent (diagnostic)

5. Please describe any abnormalities you observe, including any unexpected appearances of normal structures.
   Fill-in response, answer options: any.

Supplemental materials undergo peer review but are not copyedited, and may include typographical errors.

Crew Survey Questions

We are interested in how to improve our system: in making it easier to use and take the images you would like to see (and have the world see!). Thank you for helping that effort by answering these 5 questions:

1. Likert Scale: The x-ray system was easy to operate.
   Answer options: "strongly agree," "agree," "neutral," "disagree," and "strongly disagree"

2. Likert Scale: The Graphical User Interface was easy to use.
   Answer options: "strongly agree," "agree," "neutral," "disagree," and "strongly disagree"

3. Likert Scale: The protocol was easy to follow.
   Answer options: "strongly agree," "agree," "neutral," "disagree," and "strongly disagree"

4. Fill-in response: Please tell us a few ways we improve this system to make it easier to use.
   Answer options: any

5. Fill-in response: In the future, what would you like to see in terms of space imaging - (IE, body parts like the foot, image types like x-ray, ultrasound or MRI) - if you could choose? If you have no preference, please tell us your favorite image from this study (IE: hand, chest, forearm, pelvis, anything as long as it's of me).
   Answer options: any
